# Altered Formalin-Induced Pain and Fos Induction in the Periaqueductal Grey of Preadolescent Rats following Neonatal LPS Exposure

**DOI:** 10.1371/journal.pone.0098382

**Published:** 2014-05-30

**Authors:** Ihssane Zouikr, Morgan H. James, Erin J. Campbell, Vicki L. Clifton, Kenneth W. Beagley, Christopher V. Dayas, Deborah M. Hodgson

**Affiliations:** 1 Laboratory of Neuroimmunology, School of Psychology, University of Newcastle, Newcastle, New South Wales, Australia; 2 Neurobiology of Addiction Laboratory, School of Biomedical Sciences and Pharmacy, University of Newcastle, Newcastle, New South Wales, Australia; 3 Robinson Institute, University of Adelaide, Adelaide, South Australia, Australia; 4 Institute of Health Biomedical Innovation, Queensland University of Technology, Brisbane, Queensland, Australia; University of Medicine & Dentistry of NJ - New Jersey Medical School, United States of America

## Abstract

Animal and human studies have demonstrated that early pain experiences can produce alterations in the nociceptive systems later in life including increased sensitivity to mechanical, thermal, and chemical stimuli. However, less is known about the impact of neonatal immune challenge on future responses to noxious stimuli and the reactivity of neural substrates involved in analgesia. Here we demonstrate that rats exposed to Lipopolysaccharide (LPS; 0.05 mg/kg IP, *Salmonella enteritidis*) during postnatal day (PND) 3 and 5 displayed enhanced formalin-induced flinching but not licking following formalin injection at PND 22. This LPS-induced hyperalgesia was accompanied by distinct recruitment of supra-spinal regions involved in analgesia as indicated by significantly attenuated Fos-protein induction in the rostral dorsal periaqueductal grey (DPAG) as well as rostral and caudal axes of the ventrolateral PAG (VLPAG). Formalin injections were associated with increased Fos-protein labelling in lateral habenula (LHb) as compared to medial habenula (MHb), however the intensity of this labelling did not differ as a result of neonatal immune challenge. These data highlight the importance of neonatal immune priming in programming inflammatory pain sensitivity later in development and highlight the PAG as a possible mediator of this process.

## Introduction

Neonatal pain experiences such as hindpaw incision or inflammation are known to produce developmentally regulated changes in the nociceptive pathways, and subsequently exaggerated responses to future noxious and non-noxious stimuli [1]–[3]. Both clinical and animal studies have shown that changes in endogenous pain modulation can also occur as a consequence of neonatal inflammatory pain [4]–[6]. Although the effects of neonatal pain experiences on processing of pain later in life are well documented, the impact of neonatal exposure to mild stimuli, such as lipopolysaccharide (LPS), on subsequent inflammatory pain responses is less well understood.

Exposure to LPS during perinatal life is an established model of early life immune stress [7]–[10]. Our laboratory and others have demonstrated that neonatal LPS exposure alters immune and neuroendocrine function later in life [11]–[13]. Interestingly, we recently demonstrated that neonatal LPS exposure can also affect inflammatory pain responses, as indicated by enhanced susceptibility to formalin-induced flinching in LPS-challenged preadolescent rats [10]. We have also shown that LPS-induced behavioural hyperalgesia observed at postnatal day (PND) 22 is accompanied by increased plasma corticosterone responses and changes in the intrinsic properties of spinal dorsal horn neurons [10].

The inhibitory descending pathways travelling from the periaqueductal grey (PAG) via the dorsolateral funiculus (DLF) to the rostroventromedial medulla (RVM) and the spinal dorsal horn constitute a major anatomical circuit for the descending modulation of pain [14], [15]. This system becomes fully mature by the third week of development [16]. The PAG serves key functions in this inhibitory descending pathway including promoting analgesia [17]–[20]. For instance, formalin injection into the hindpaw of infant and adult rats is associated with Fos expression in the PAG [21], [22]. Further, PAG stimulation or an injection of morphine prior to formalin injection attenuated formalin-induced nociception [23]. Anatomically, the PAG is organized into longitudinal columns, namely dorsal PAG (DPAG), lateral PAG (LPAG) and ventrolateral PAG (VLPAG) [24]. Whereas electrical, opiate, or amino acid stimulation of the ventral PAG elicits opioid-dependent analgesia, electrical or glutamatergic stimulation of the DPAG or LPAG induces analgesia that is not blocked by opioid antagonist (e.g. naloxone) [24]–[27]. One structure that provides significant input onto the PAG to mediate antinociception is the habenula, particularly its lateral subdivision (LHb) [28], [29]. Morphological and electrophysiological studies have indicated that the habenula receives and modulates noxious inputs [30], [31]. For example, electrical stimulation or microinjection of morphine into the habenula induces analgesia in the formalin test [32], [33] and an injection of formalin into the hindpaw of rats is reported to elicit the expression of Fos-protein within the LHb [34]. Formalin injections are also associated with increased Fos-protein expression in stress-sensitive regions, including the paraventricular thalamus (PVT) [22].

Clearly, early life events are important in shaping the nociceptive circuitry. We have previously demonstrated that neonatal LPS exposure exerts developmentally regulated changes in formalin-induced behaviours, corticosterone levels, and dorsal horn neuronal properties with pronounced changes observed particularly at PND 22 [10]. In the present study, we aim to determine the supra-spinal changes associated with the neonatal immune challenge in PND 22 rats. Specifically, whether the increased flinching responses observed in preadolescent rats treated with LPS as neonates is associated with altered neuronal activity within specific subregions of the PAG, an essential substrate for opioid-induced analgesia [35], [36]. Our hypothesis is that exposure to LPS during the first postnatal week, when the descending inhibitory systems are still functionally immature [16], will alter the neuronal activity within the PAG and subsequently alter the behavioural responses to formalin injection at PND 22.

## Materials and Methods

### Animals and Ethics Statement

Five experimentally naïve female Wistar rats were obtained from the University of Newcastle Animal House and allowed one week acclimatisation, after which two males were introduced to their cages. The males were removed after two weeks and dams were housed individually in custom designed polycarbonate-perspex home boxes (43.5 cm×28 cm×12.5 cm; Mascot Wire Works, Sydney, Australia). Mating occurred at the University of Newcastle Psychology vivarium and resulted in a total of 68 offspring, from which a subset of males (n = 13) was selected for use in this study. A maximum of three pups per litter were assigned to each group and for each experimental condition; animals were distributed as evenly as possible from all litters used per treatment to avoid potential litter effects. Until their allocated testing day, rats were maintained in a temperature (21±1°C) and humidity (60%) controlled environment, under a 12 h/12 h light-dark cycle (light on 06∶00 h) with food (Rat and Mouse Pellets, Glen Forest, Western Australia) and water available *ad libitum*. All experiments were carried out in accordance with the National Health and Medical Research Council Australian Code of Practice for the care and use of animals for scientific practice. All procedures were reviewed and approved by the University of Newcastle Ethics committee (Ethics approval no. A-2010-127).

### Neonatal Endotoxin Exposure

At PND 3 and 5 (birth considered as PND 1), pups were briefly removed from their home cages, weighed and administered intraperitoneally (i.p) with either LPS (from *Salmonella enterica*, serotype *enteritidis*; Sigma-Aldrich, USA, dissolved in 20 µl sterile pyrogen-free saline, 0.05 mg/kg) or an equivalent volume of sterile saline (Livingstone International, Australia). All injections were made between 9∶00 am and 10∶00 am. This model has been previously used in our [37], [38] and other laboratories [12], [39] with the dosage and timing having been demonstrated to induce a rapid sustained febrile response, but no mortality.

### Formalin Testing Procedure

Unlike traditional tests of nociception such as the tail flick and hot plate tests which investigate acute pain, the formalin test is a widely accepted model of persistent pain and more closely resembles clinical cases of chronic pain [40]. Injection of formalin into the hindpaw of rodents produces a characteristic biphasic response of flinching and licking of the injected paw, with an early phase (0–5 min) and a late phase lasting up to 90 min [41], [42].

The formalin solution was prepared using Formaldehyde (36.5%–38%; Biolab Ltd, Victoria, Australia) and preservative-free saline (Sodium chloride Injection BP 0.9%, Pfizer, Australia). At PND 22, all rats (neonatal saline: n = 6; neonatal LPS: n = 7) underwent a subcutaneous injection of 1.1% formalin into the plantar surface of the left hindpaw using a 31 G needle (10 µl). The choice of formalin concentration range, volume and site of injection was based on our previous work [42]. Rats were tested in transparent Plexiglas boxes (30 cm (w)×30 cm (l)×30 cm (h)). A mirror was mounted 45° beneath the floor to allow for an unobstructed view of the paws and a camera was mounted to record the behavioural responses from the reflection of the mirror. Behavioural recording was done on a DVD recorder for one-hour post formalin injection. Flinching and licking were scored based on the method of Wheeler-Aceto and Cowan [43] and was carried out by a trained experimenter blind to treatment condition of each animal. The one-hour of behavioural recording was divided into an early phase (the first 5 min) and a late phase (10 to 60 min) during which the frequency of flinching (paw lifting or shaking) as well as the duration licking the injected paw (in seconds) was scored. PND 22 rats were tested at room temperature and at this age, an acclimation period was not required as during this period of development rats are still unable to recognize and interact with the environment [44].

No saline-injected rats were included in this study since it has been previously shown that rats subjected to a subcutaneous injection of saline into the plantar surface of the hindpaw do not display flinching or licking behaviour when tested during the first three postnatal weeks and adulthood [45]–[47]. Prior spinal cord Fos studies showed that infant rats receiving less than 20 µl saline demonstrated no Fos labelling [48].

### Perfusion, Brain Collection and Immunohistochemistry

Rats were deeply anaesthetized with an overdose of Lethabarb (2 mg/kg i.p; Virbac, Pty. Ltd, Milperra, Australia) one and a half hours following the formalin test. This time point was selected as Fos-protein expression has been shown to peak at 1.5–2 hrs following stimuli exposure [49], [50]. Animals were transcardially perfused with 150 mls of normal saline followed by 500 mls of 4% paraformaldehyde (pH 9.5). Brains were removed, postfixed and cryoprotected (24 hours, at 4°C) in the same fixative solution with the addition of 15% sucrose. Brains were then stored in 15% sucrose in 0.1 M phosphate buffer (pH 7.4 at 4°C). Serial coronal sections of the rostral forebrain (40 µm) and caudal midbrain (50 µm) were cut on a freezing microtome (Leica SM 2000R, Leica Biosystems, Germany). A 1-in-4 series of brain sections from the habenula (lateral and medial sections, bregma −2.16 to −3.60) and the PVT (bregma −2.16 to −3.60), and a 1-in-5 series of the rostral (bregma −7.64), medial (bregma −8.0) and caudal (bregma −8.3) PAG [51] were processed for immunohistochemical detection of Fos-protein (72 h, 1∶10000, rabbit polyclonal, Santa Cruz Biotechnology, CA, USA) as described previously [52], [53]. Sections were then incubated in a secondary antibody biotinylated anti-rabbit (2 hours, 1∶300 donkey anti-rabbit, Jackson ImmunoResearch, PA, USA). Finally, sections were incubated in diaminobenzodine (DAB) in 2% filtered nickel sulphate (NiSO_4_) for 15 minutes before glucose oxidase (0.2 µL per mL of solution) was added to visualise Fos-protein.

Fos-positive cell counts were determined by creating boundaries around each brain structure. The VLPAG boundary did not include the raphe nuclei. After selecting the region of interest, a thresholding procedure was used for Fos expression. Fos counts were quantified using MetaMorph Imaging System Software (Version 7.5; MDS Analytical Technologies) under a 10x microscopic objective (Olympus CX40).

## Statistical Analysis

Data analysis was carried out using the Statistical Package for the Social Sciences for Windows, version 20 (SPSS). A Linear Mixed Model (LMM) was applied to analyse the behavioural, and Fos-protein immunohistochemistry data (For more details about LMM, see [42]). For behavioural responses, the area under the curve (AUC) which was the sum of flinching or licking responses from 10 to 60 min was calculated and analysis was carried out using an ANCOVA with litter size as a covariate variable. Post-hoc analyses (two-tailed) were carried out using Least Significant Differences (LSD) tests. An alpha value of 0.05 was adopted for all tests.

## Results

### Formalin Responses in Preadolescent Rats: Neonatal Immune Challenge Alters Formalin-induced Nociception in both the Early and Late Phase

**Figure 1:**
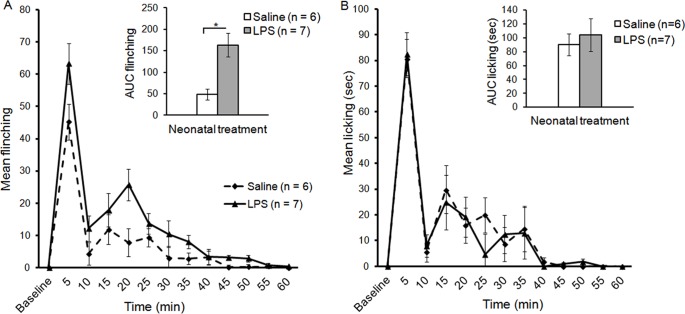
Neonatal LPS exposure enhances formalin-induced nociception in preadolescent rats. Time course of flinching (A) and licking (B) responses following an injection of 1.1% formalin (mean ± SEM). AUC: the Area Under the Curve.

The characteristic biphasic response was observed in preadolescent rats in both treatment groups in flinching and licking of the injected paw. However, neonatal LPS-treated rats displayed higher flinching during the early and late phase. LMM analysis of flinching responses revealed a significant two way interaction of Time and Treatment [*F*(7,11) = 8.02, *p*<.01] implying that neonatal LPS treatment altered the inflammatory pain response in preadolescent rats. Pairwise comparisons revealed that LPS treated rats displayed significantly higher flinching responses during the late phase at 20 min post formalin injection when compared to their matched saline control group (*p*<.05; Figure 1A). Pairwise comparisons also revealed that during the early phase (first 5 min), LPS-treated rats had significantly higher flinching responses compared to saline (*p*<.05). Moreover, analysis of the AUC during the late phase revealed that LPS treated rats had significantly higher flinching responses compared to the saline group [*F*(1,11) = 5.25, *p*<.05; Figure 1A]. LMM analysis revealed no significant differences in licking responses between neonatally saline or LPS-treated rats in both the early and late phase of the formalin test (Figure 1B).

### Formalin-induced Fos-protein Expression in Brain Areas Involved in Stress and Analgesia

**Figure 2:**
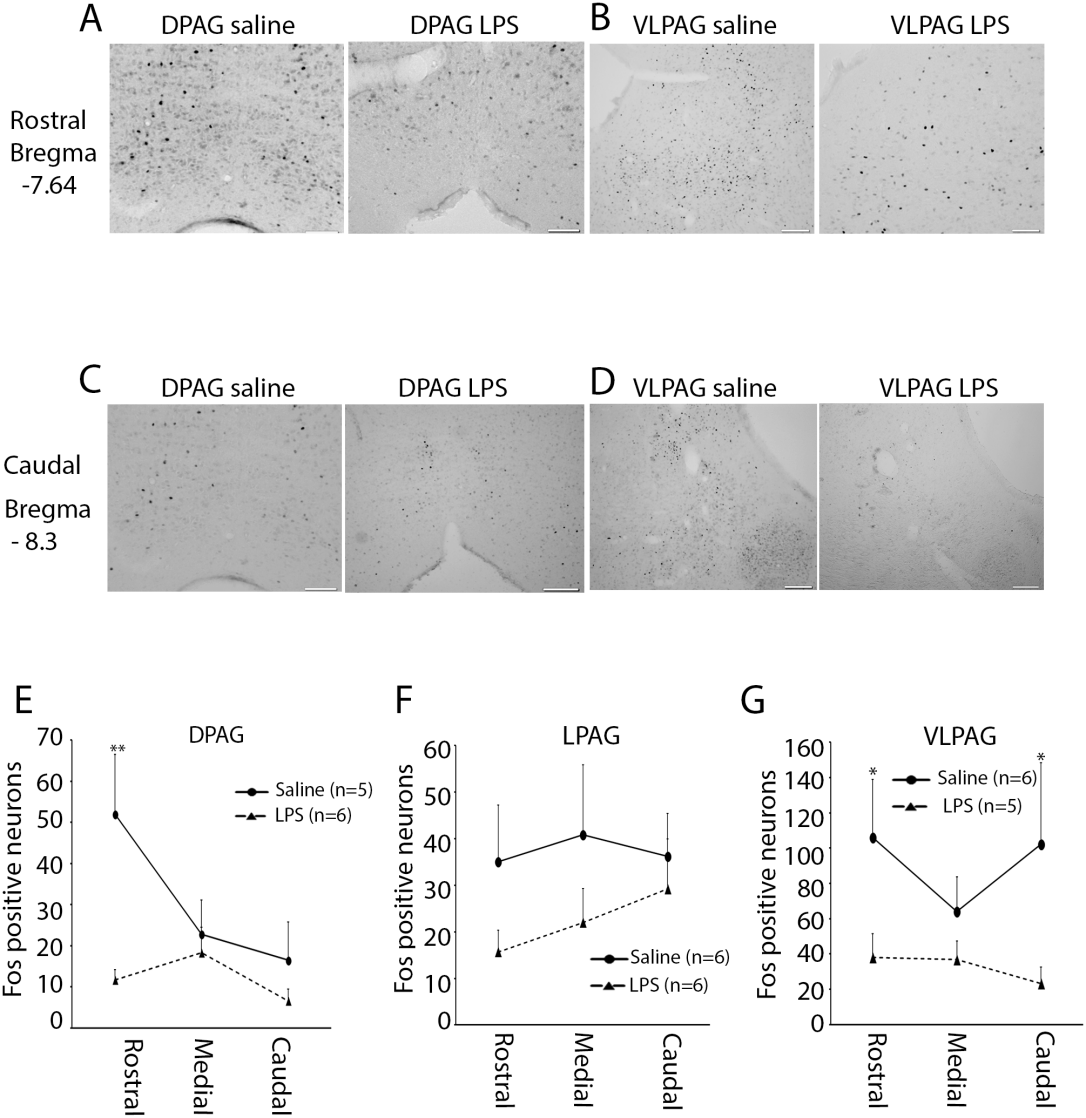
Fos immunoreactivity in the midbrain periaqueductal grey (PAG) following a neonatal immune challenge and subsequent inflammatory pain. Representative examples illustrate the distribution of Fos-positive nuclei in the dorsal and ventrolateral PAG (DPAG, VLPAG, respectively) at the rostral (A & B) and caudal (C & D) axes following formalin injection in preadolescent rats. (E) Quantification of Fos-positive nuclei in DPAG between neonatal saline and LPS-treated rats. (F) Quantification of Fos-positive nuclei in lateral PAG (LPAG). (G) Quantification of Fos-positive nuclei in VLPAG. Data are presented as mean ± SEM. *p<.05; **p<.01. Scale bar = 100 µm.

Fos-protein expression in the PAG was examined in the three main subdivisions (DPAG; LPAG; and VLPAG) and across three rostrocaudal axes (rostral, medial, and caudal). In the DPAG, LMM analysis revealed a significant effect of treatment [*F*(1,25) = 7.52; *p*<.05] within the DPAG at the rostral level. Pairwise comparisons revealed that neonatal saline-treated rats displayed significantly higher numbers of Fos-positive cells compared to neonatal LPS-treated rats following formalin injection (*p*<.01). No significant differences were found in the medial and caudal part of the PAG (Figure 2E, Table 1).

**Table 1:** Fos expression in the periaqueductal grey (PAG).

Treatment	Axis	DPAG	LPAG	VLPAG
Sal/formalin	Rostral	52.0±14.5	35.0±12.2	106.0±33.1
	Medial	22.8±8.3	40.8±14.9	64.0±19.7
	Caudal	16.5±10.2	36.2±9.1	102.2±46.3
LPS/formalin	Rostral	11.6±2.4	15.6±4.6	38.0±13.6
	Medial	18.3±6.0	22.0±7.2	36.8±10.6
	Caudal	6.6±3.1	29.2±4.9	23.2±9.3

Note: Data are presented as mean number of Fos-positive cells per section ± SEM.

No significant differences were found in terms of Fos-positive nuclei on the ipsilateral versus contralateral side of either the LPAG or the VLPAG, and therefore these data were combined. In the LPAG, LMM analysis revealed a strong trend towards decreased Fos-positive neurons in LPS-treated rats, however this did not reach significance (*p* = .062; Figure 2F). In contrast, there was a significant main effect of treatment on the induction of Fos-protein in both the rostral and caudal VLPAG [*F*(1,34) = 5.71; *p*<.05; *F*(1,34) = 8.39; *p*<.01, respectively]. Pairwise comparisons revealed that neonatal saline-treated rats exhibited significantly higher Fos-positive neurons compared to LPS animals in the rostral (*p* = .05) and caudal (*p*<.05) VLPAG (Figure 2G, Table 1).

**Figure 3:**
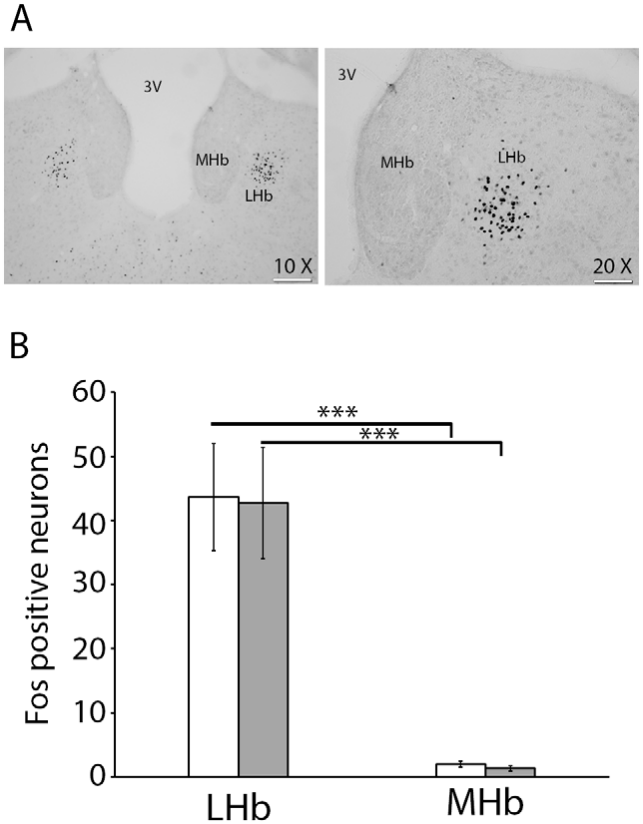
Fos immunoreactivity in the habenula following a neonatal immune challenge and subsequent inflammatory pain. (A) Representative examples illustrate the distribution of Fos-positive nuclei in the lateral and medial habenula (LHb & MHb, respectively). (B) Quantification of Fos-positive nuclei in LHb and MHb in neonatal saline (white bar) and LPS-treated rats (grey bar) after formalin injection in preadolescent rats. 3V: third ventricle. ***p<.001. Scale bar = 100 µm for the 10x microscopic objective and 200 µm for 20x microscopic objective.

Bilateral Fos-protein labelling was observed in the LHb and medial Habenula (MHb). No significant differences were observed in Fos-like immunoreactivity in the ipsilateral versus contralateral sides of formalin injection, and therefore data from both sides were combined. LMM analysis revealed a significant main effect of subregion [*F*(1,18) = 46.17; *p*<.001] implying that the LHb and MHb showed different pattern of Fos activation. Pairwise comparisons indicated that regardless of the neonatal challenge, significantly greater levels of Fos-protein expression were observed in the LHb compared to the MHb following formalin injection (*p*<.001; Figure 3A & 3B).

The PVT is known to express Fos following formalin injection into the hindpaw in rats [22]. There was a trend towards increased levels of Fos immunoreactivity in the PVT in neonatal saline-treated animals compared to LPS-treated animals, however this failed to reach significance (*p* = .175) (Table 2).

**Table 2:** Fos expression in the paraventricular nucleus of the thalamus (PVT) and the lateral and medial habenula (LHb & MHb).

Treatment	PVT	LHb	MHb
Sal/formalin	22.1±6.0	43.6±8.3	1.9±0.4
LPS/formalin	14.3±2.1	42.7±8.6	1.2±0.3

Note: Data are presented as mean number of Fos-positive cells per section ± SEM.

## Discussion

The current study demonstrates that exposure to an immune challenge during the neonatal period is accompanied by enhanced behavioural responses to formalin in preadolescent rats. We observed increased susceptibility of preadolescent rats to formalin-induced flinching but not licking behaviours in LPS-treated rats. This LPS-induced hyperalgesia was associated with distinct recruitment of supra-spinal structures as indicated by significantly attenuated Fos-protein expression in the rostral DPAG as well as rostral and caudal axes of the VLPAG.

### Neonatal Exposure to LPS Alters Flinching but not Licking Responses in Preadolescent Rats

The nociceptive system undergoes fine-tuning and maturation during the first postnatal weeks of development [54]. While C-fibers are present within the spinal cord prenatally, they become functional and respond to noxious stimuli only during the second postnatal week. For instance, the ability of C-fibers to produce neurogenic oedema appears only at PND 11–14 [55]. Moreover, application of mustard oil, a specific C-fibers irritant, to the hindlimb skin does not induce Fos-protein expression in the spinal cord until the second week of development [56]. These findings suggest that the nociceptive circuitry is highly malleable during early postnatal life. Therefore, exposure to a physiological insult such as LPS during the first weeks of postnatal life, where the neurocircuitry underlying nociception undergoes significant plasticity, is likely to interfere with the normal developmental trajectory of the nociceptive system, leading to altered behavioural and neuronal responses following exposure to noxious stimuli later in development.

The current study demonstrated that neonatal LPS exposure produced increased flinching but not licking during both phases of the formalin test. These findings are in accordance with our previous work whereby neonatal LPS exposure was able to alter flinching but not licking patterns in preadolescent rats [10]. Flinching and licking are mediated by distinct neural pathways. Whilst the spontaneous rhythmicity of flinching behaviour suggests this behaviour is spinally modulated, the complex movements involved in licking behaviour suggest it engages supraspinal modulation. Indeed, intrathecal injection of Lidocaine into the lumbar spinal cord prior to formalin injection abolishes flinching response during the late phase [57]. However, transection of the spinal cord at the mid-thoracic level has been shown to have no effect on formalin-induced flinching [57], but completely abolishes formalin-induced licking [43]. The findings reported in these animal studies suggest that the generation of flinching and licking behaviours result from the activation of distinct neural pathways. Exposure to an immune challenge is thus able to alter either the spinal or supraspinal circuitry leading to alteration in the intensity and profile of the biphasic formalin response later in development. Together, the current findings suggest that neonatal exposure to LPS enhances the sensitivity of preadolescent rats to inflammatory noxious stimuli (i.e. hyperalgesia). This is in accordance with a recent study demonstrating that rats challenged with LPS at PND 14 displayed enhanced mechanical and thermal nociception when tested in adulthood (8 to 12 weeks old) [58]. In this study a subsequent exposure to LPS in adulthood failed to alter the behavioural responses to mechanical and thermal stimuli [58]. These data suggest that a single immune challenge is sufficient to disrupt nociception later in life. The current findings add to the literature by demonstrating that a neonatal immune challenge can also alter inflammatory nociception as indicated by enhanced formalin-induced flinching in preadolescent rats.

### Neonatal LPS Exposure Results in Distinct Recruitment of Supra-spinal Structures Involved in Analgesia following Formalin Injection

Electrical or chemical stimulation of the PAG produces profound analgesia in rats [18], [20], [25], [36], [59], [60]. The PAG is organized into longitudinal columns [24], [61], with a dorsal, lateral, and ventrolateral column. The current study demonstrates that in response to a neonatal immune challenge and subsequent inflammatory pain at PND 22, preadolescent rats exhibited distinct patterns of Fos-protein expression within these columns. At PND 22, following formalin injection, LPS-challenged rats exhibited significantly attenuated levels of Fos-protein in both rostral DPAG and rostral and caudal VLPAG compared to saline-challenged rats. Interestingly, although there was a strong trend towards reduced Fos-protein expression in the LPAG in LPS-treated rats, this did not reach statistical significance, possibly due to the lack of power in our analyses. These findings are in agreement with recent observations showing significantly increased Fos immunolabelling in the caudal part of the DPAG and VLPAG following formalin injection in PND 14 rats [22]. Electrical or opiate stimulation of the DPAG and VLPAG has been previously reported to induce analgesia in infant and adult rats [62]–[64]. For instance, injection of the µ-opiate agonist, DAMGO into both ventral and dorsal PAG was associated with enhanced paw withdrawal latency to a noxious thermal stimulus (47°C water) in 3, 10, and 14 day-old rats [63]. Since stimulation of both the VLPAG and DPAG mediates analgesia [25], [65], the reduction in Fos-labelling within the DPAG and VLPAG of LPS-challenged rats is likely associated with the observed hyperalgesia in these animals following the formalin injection.

In addition to the PAG, the LHb has also been demonstrated to play an important role in mediating analgesia in the formalin test [32], [33]. Further, increased neuronal depolarization was observed in the LHb in response to noxious peripheral stimulation [30]. Importantly, these effects appear to be specific to the LHb, as adult rats injected with formalin have been previously shown to display increased Fos labelling in the LHb but not the MHb [34]. Moreover, electrical stimulation of the LHb but not the MHb in freely moving rats has been shown to evoke naloxone-reversible analgesia in the tail flick test [66]. Together, these findings suggest that compared to the MHb, neurons in the LHb respond specifically to noxious stimulations. This is consistent with our findings where formalin injection into the hindpaw of preadolescent rats was associated with increased Fos-protein labelling in the LHb as compared to MHb. However, in the current study, the intensity of Fos-protein expression was not affected by neonatal immune challenge. It is however possible that formalin injection resulted in maximal Fos-protein expression in LHb across both groups, thereby preventing the identification of a treatment effect.

Morphological studies have revealed the existence of afferent and efferent connections between the habenula and the PAG [28], [34], [67], [68]. The habenula constitutes an important relay in the descending pathway to the PAG to subserve antinociception [69], [70]. Administration of morphine into the habenula of a rabbit produced a marked increase in the withdrawal reflex latency to radiant heat [69]. This antinociception was dose-dependently attenuated by intra-PAG administration of the opiate antagonist naloxone [69]. Furthermore, the antinociception induced by intra-habenula injection of morphine, was attenuated by muscimol (a GABA receptor agonist) injected into the PAG [69]. These data suggest that morphine can act on the habenula to activate a neural descending pathway projecting to the PAG to mediate analgesia. This pathway seems to implicate the release of endogenous opioid peptides and GABA. Of particular interest, stimulation of the LHb was reported to inhibit the unit discharges of nociceptive-specific neurons within the PAG [71]. The findings reported in these animal studies are consistent with a recent human study that revealed, using fMRI technique, an interrelated activity of habenula and PAG following noxious thermal stimulation [70].

Since no significant differences were observed in the LHb, it is possible that an alternate structure innervated the PAG to mediate the decreased behavioural responses to formalin observed in control animals. Another possibility is that the analgesia observed in control animals could be attributed to PAG activation alone. Further research is required to examine these possibilities.

## Conclusion

The ability to perceive and respond to noxious stimuli is critical for survival. Equally important is the recruitment of supra-spinal structures involved in analgesia to reduce the suffering associated with pain. The current study importantly demonstrates that neonatal exposure to LPS results in decreased neuronal activation within the PAG following exposure to a noxious inflammatory stimulus in preadolescence. We have also demonstrated that neonatal exposure to LPS produces altered behavioural responses to formalin injection in preadolescent rats. These findings highlight the importance of neonatal immune challenge in programming behavioural and supra-spinal responses to inflammatory pain later in development.
